# Supplementary figures and images for: Astrocytes influence medulloblastoma phenotypes and CD133 surface expression
Source: PLoS One. 2020 Jul 6;15(7):e0235852. doi: 10.1371/journal.pone.0235852 (PMC7337293; doi:10.1371/journal.pone.0235852)

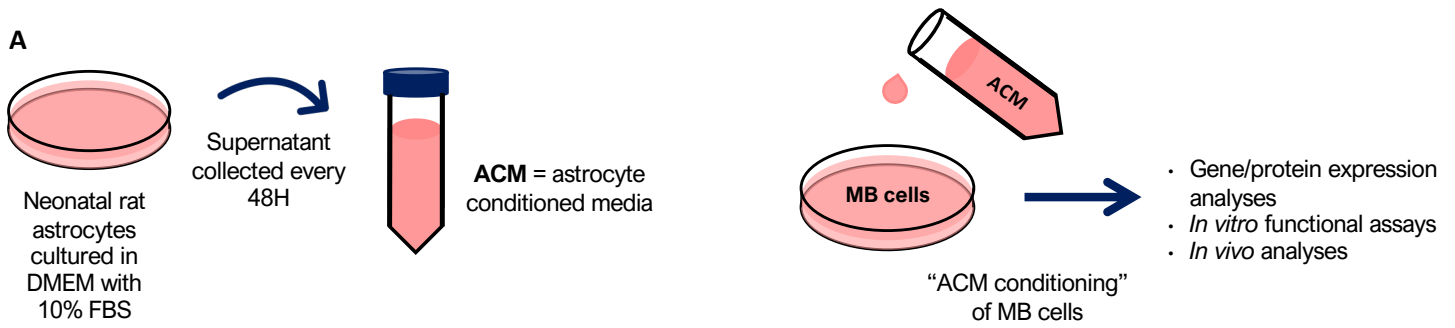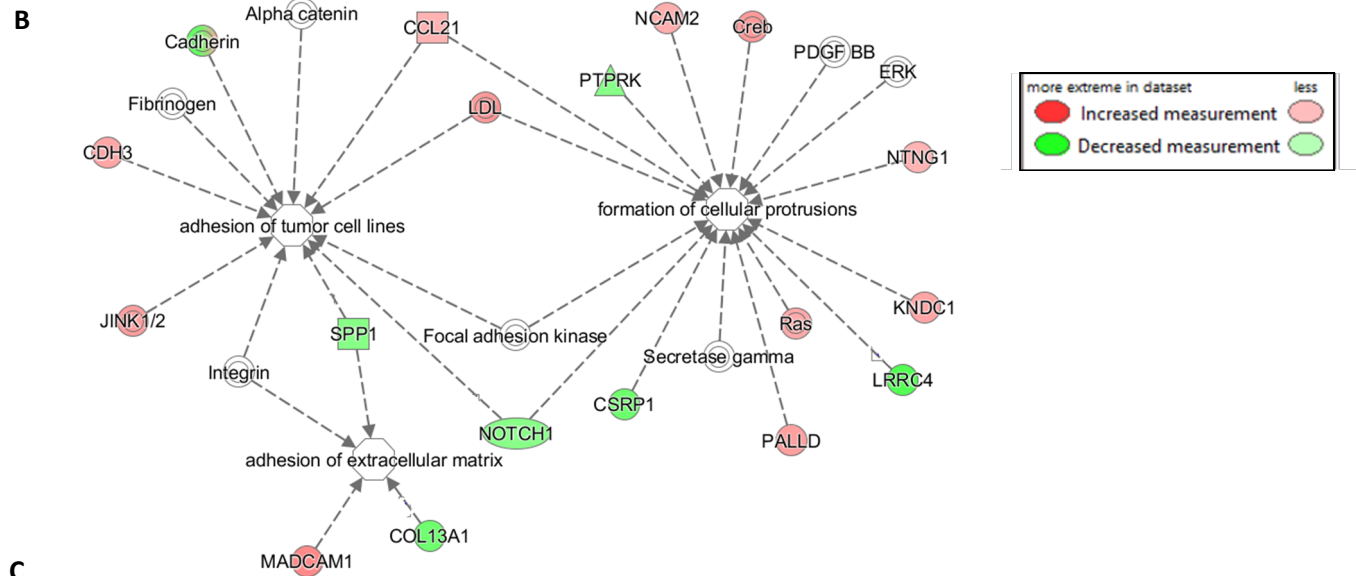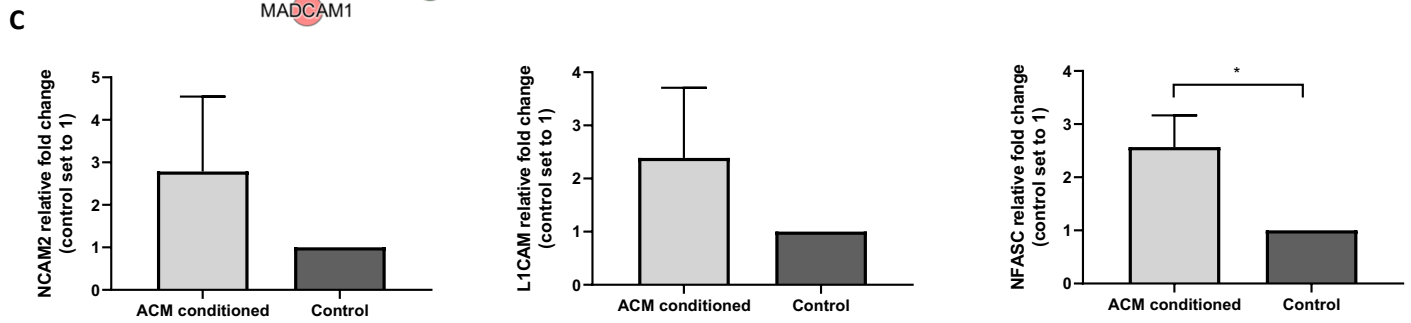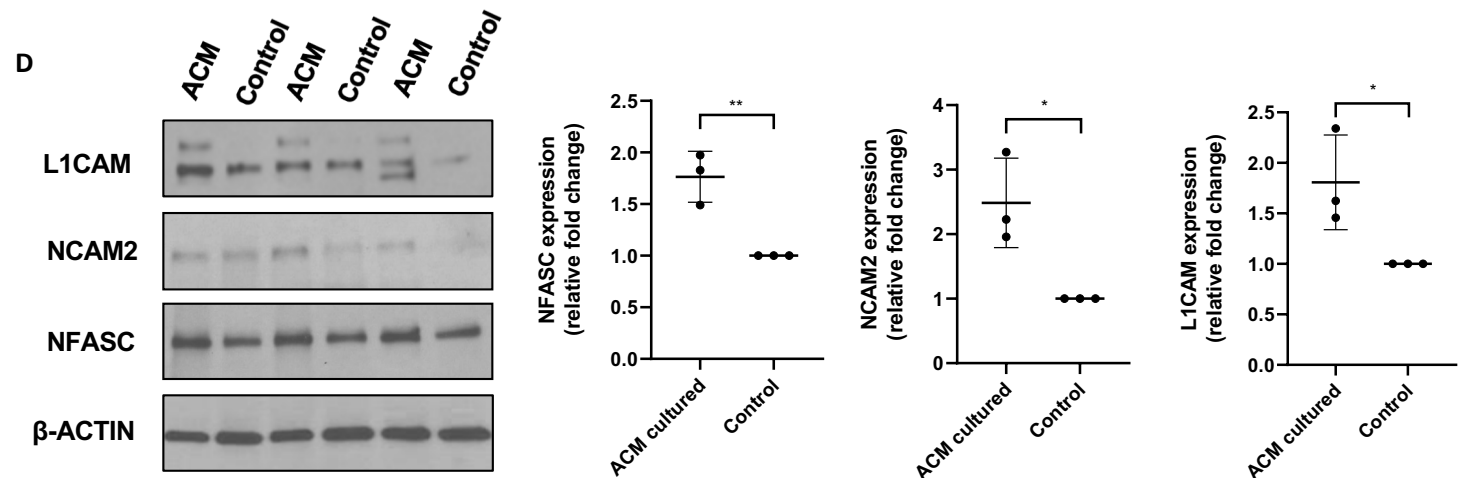

Supplement: S1 Fig — (A) depicts the ACM collection and treatment of MB cells in a pictorial format. (B) Graphic depiction of adhesion pathways predicted to be altered in ACM cultured Daoy cells based on the microarray data. Image generated using Ingenuity Pathway Analysis (Qiagen) software. (C) qRT-PCR normalized fold change compared to control (DMEM, set to 1) for three adhesion target genes (NFASC, L1CAM and NCAM2) identified as significantly changed in the microarray analysis. (D) Western blot analysis of NFASC, L1CAM, and NCAM2 in the ACM-conditioned and DMEM-conditioned MB cell lysates. β-actin served as a loading control (left). Quantification of relative fold change measured by the densitometry of western blot bands and normalized to β-actin (DMEM condition set to 1) (right). Quantified values expressed as mean +/- SD. *p<0.05, **p<0.01, ***p<0.001, ****p<0.0001. (PDF) [file pone.0235852.s001.pdf]

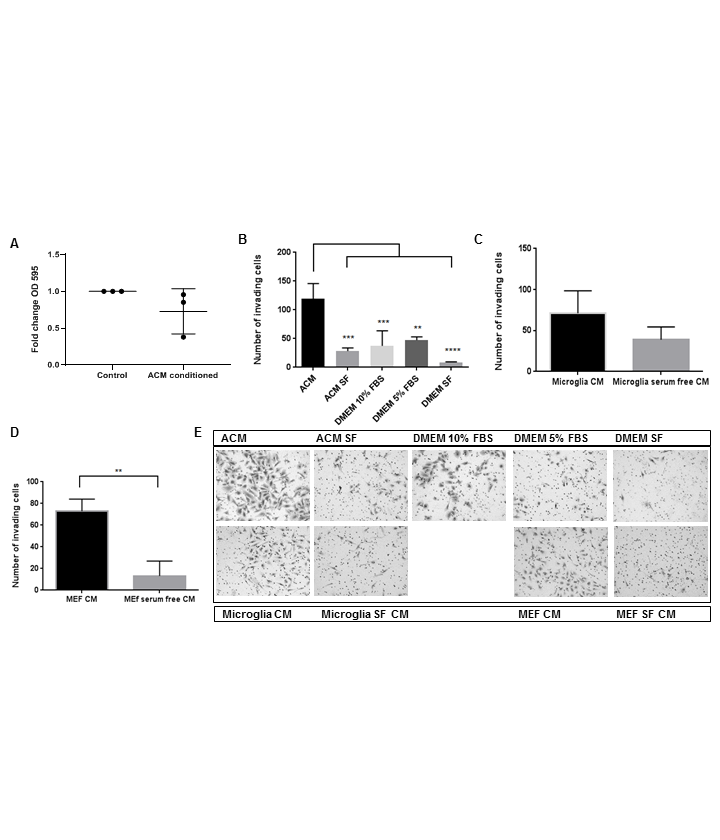

Supplement: S2 Fig — (A-B) Fold change of the number of adhered cells per well, measured by dissolving the stained cells and reading optical density at 595 nm in ACM conditioned cells compared to control (DMEM, set to 1). (B-D) Average number of invaded cells per image, counted on the lower surface of Boyden chambers, with the respective media in the lower well during incubation. Panel C is microglia conditioned media (CM) cells and (D) is mouse embryonic fibroblast (MEF) media conditioning. (E) Representative images of invaded cells on the lower surface of the Boyden chambers in response to various conditioned media are shown. Quantified values expressed as mean +/- SD. **p<0.01, ***p<0.001, ****p<0.0001. (TIF) [file pone.0235852.s002.tif]
