# Supplementary material for: Astrocytes influence medulloblastoma phenotypes and CD133 surface expression
Source: PLoS One. 2020 Jul 6;15(7):e0235852. doi: 10.1371/journal.pone.0235852 (PMC7337293; doi:10.1371/journal.pone.0235852)
Supplement: S1 Raw images — (PDF) [file pone.0235852.s004.pdf]

Figure 3E

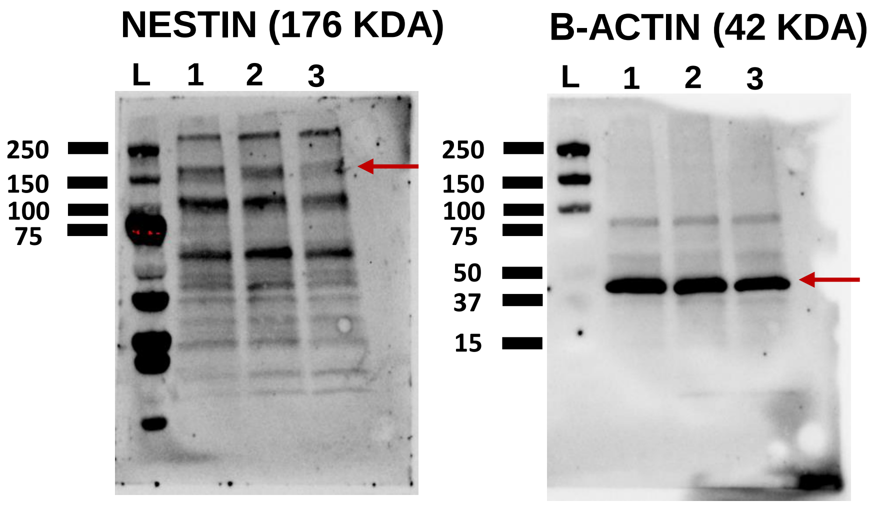

L – ladder
1 – DMEM:F12 NS 48H
2 – ACM NS 48H
3 – DMEM NS 48H

Figure 3E (not pictured)

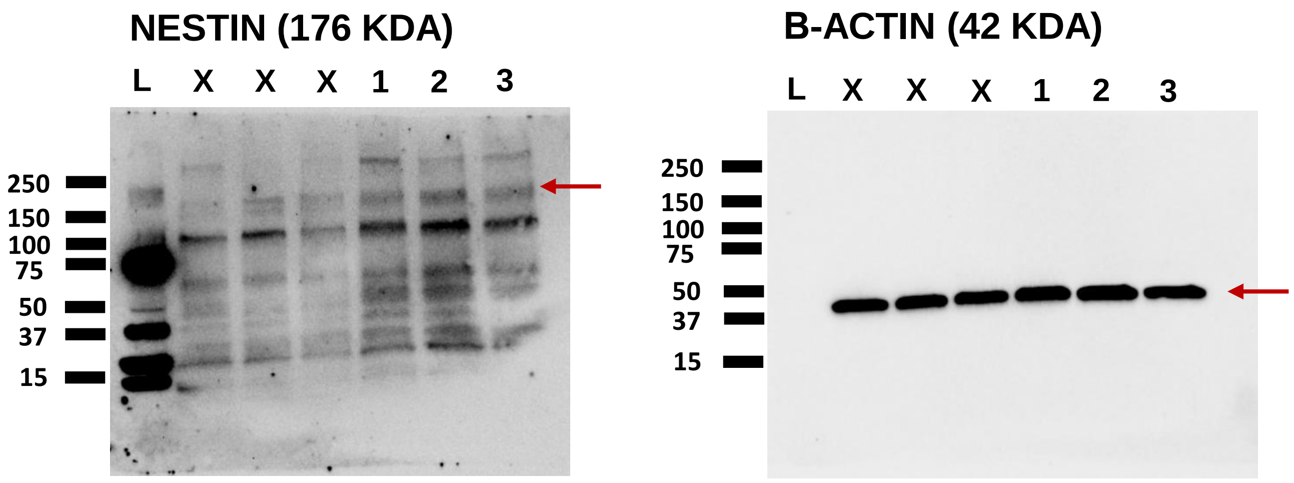

L – ladder
1 – DMEM NS 48H
2 – DMEM:F12 NS 48H
3 – ACM NS 48H

Figure 3E (not pictured)

NESTIN (176 KDA)

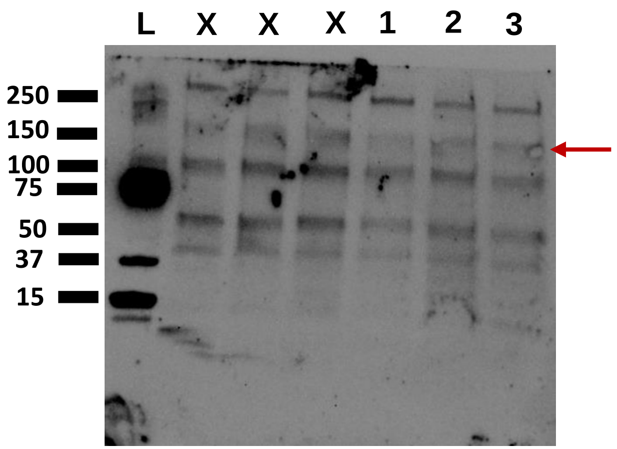

B-ACTIN (42 KDA)

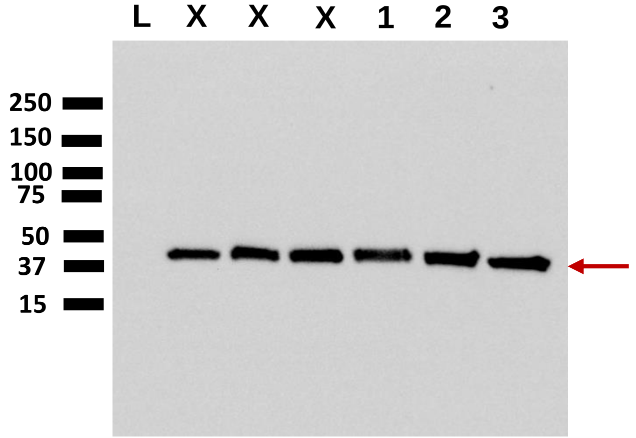

L - ladder
1 – DMEM NS 48H
2 – DMEM:F12 NS 48H
3 – ACM NS 48H

Figure 3F

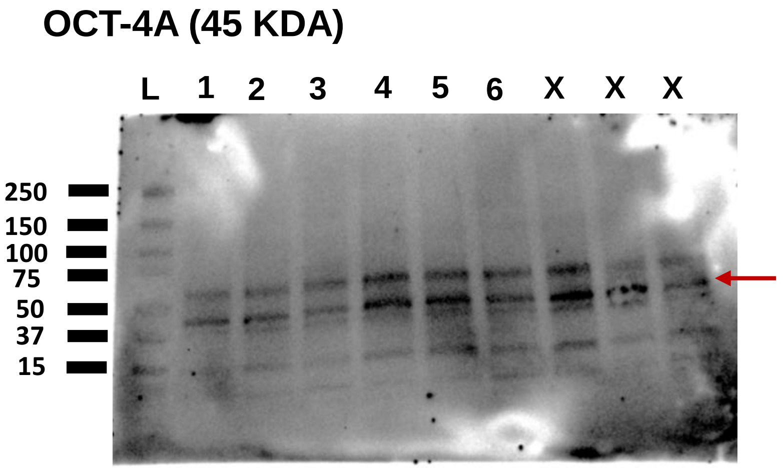

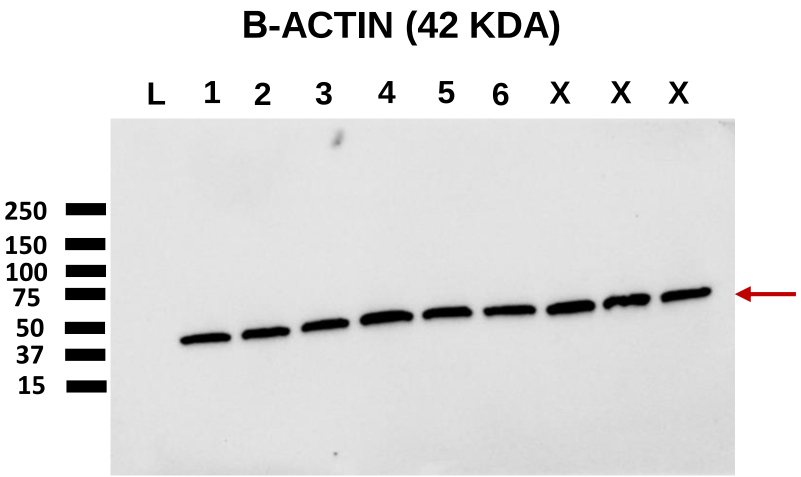

L – ladder
1 – DMEM:F12 NS 48H
2 – ACM NS 48H
3 – DMEM NS 48H
4 – DMEM:F12 NS 48H
5 – ACM NS 48H
6 – DMEM NS 48 H

Figure 3F (not pictured)

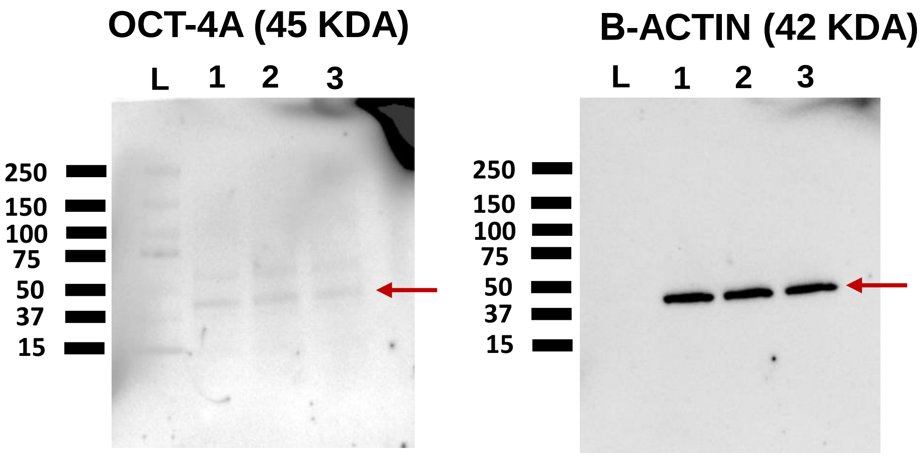

L – ladder
1 – DMEM:F12 NS 48H
2 – ACM NS 48H
3 – DMEM NS 48H

Figure 3G

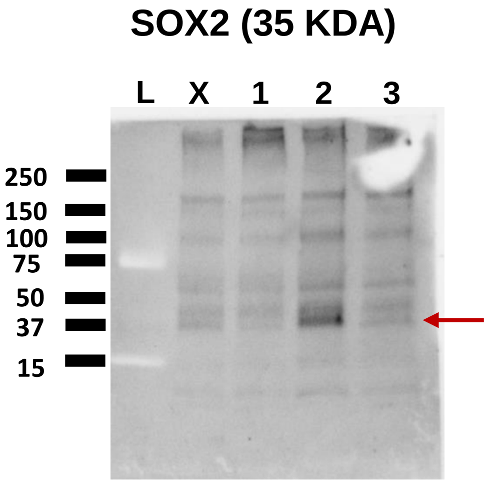

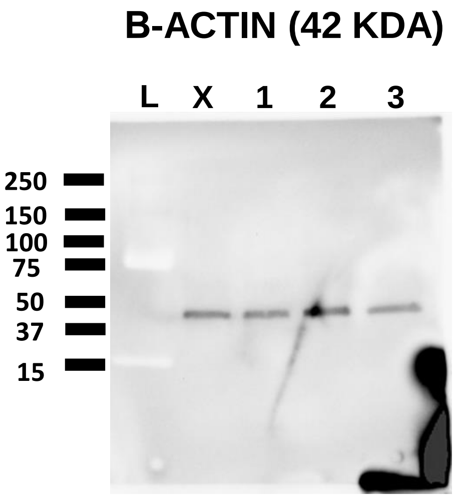

L – ladder
1 – DMEM NS 48H
2 – DMEM:F12 NS 48H
3 – ACM NS 48H

Figure 3G (not pictured)

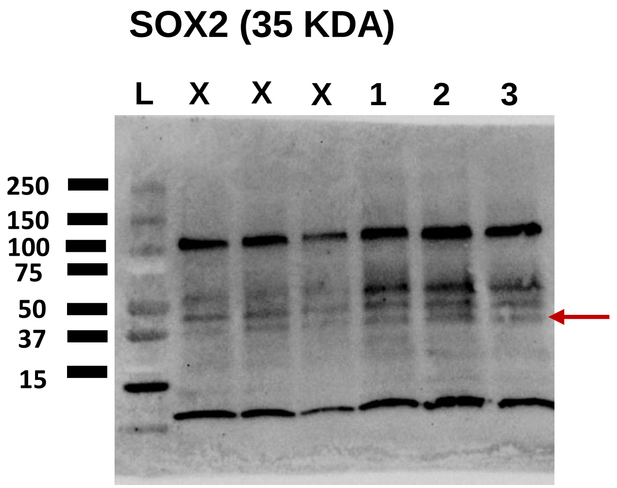

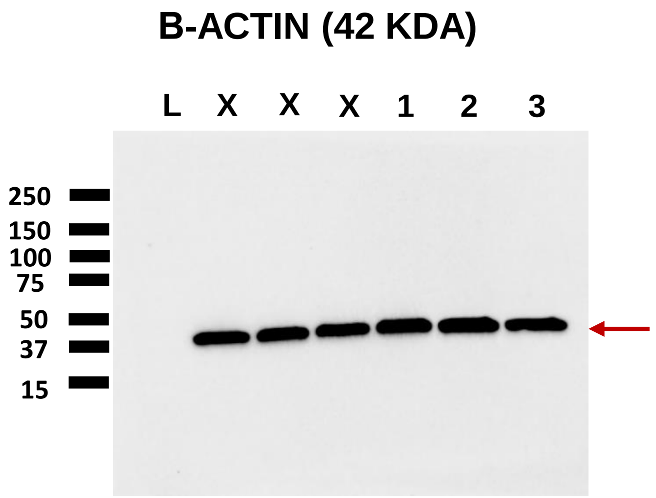

L – ladder
1 – DMEM NS 48H
2 – DMEM:F12 NS 48H
3 – ACM NS 48H

Figure 3G (not pictured)

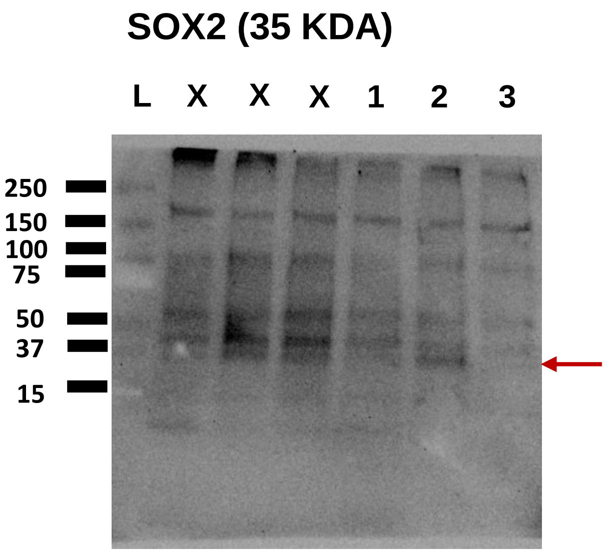

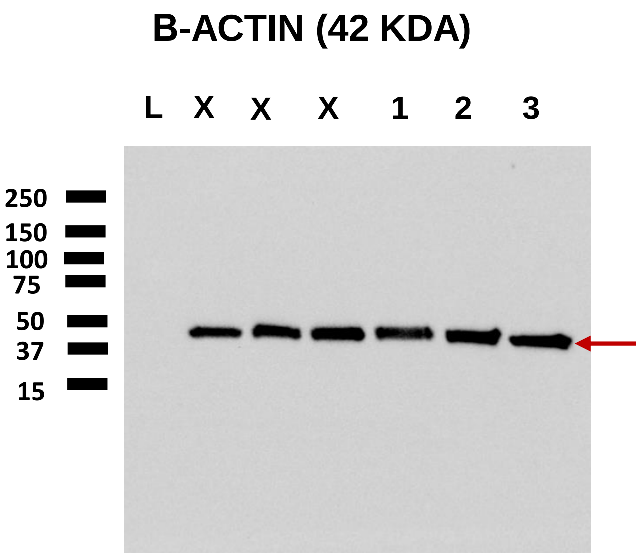

L – ladder
1 – DMEM NS 48H
2 – DMEM:F12 NS 48H
3 – ACM NS 48H

Figure 4A

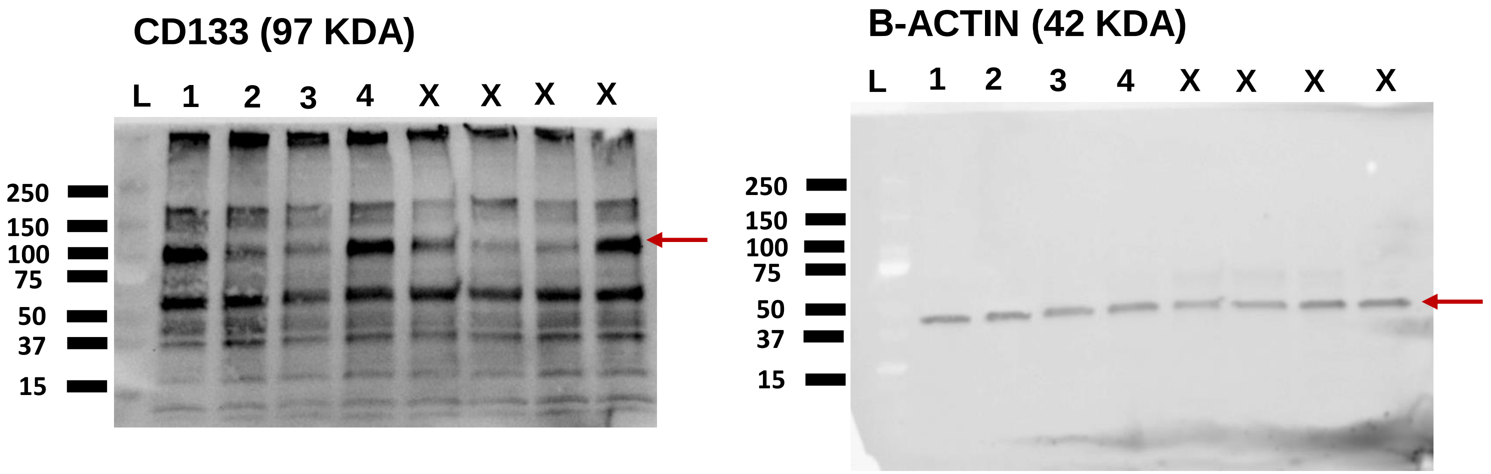

L – ladder
1 – No transfection
2 – *siCD133-1* (10nM)
3 – *siCD133-2* (10nM)
4 – *Scramble* (10nM)

Figure 4A (not pictured)

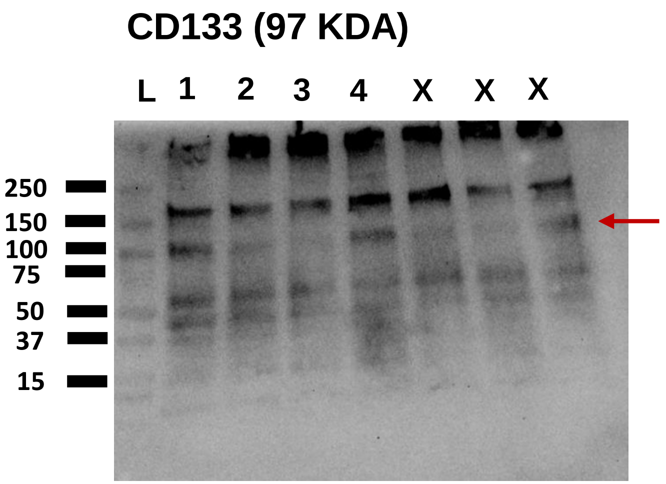

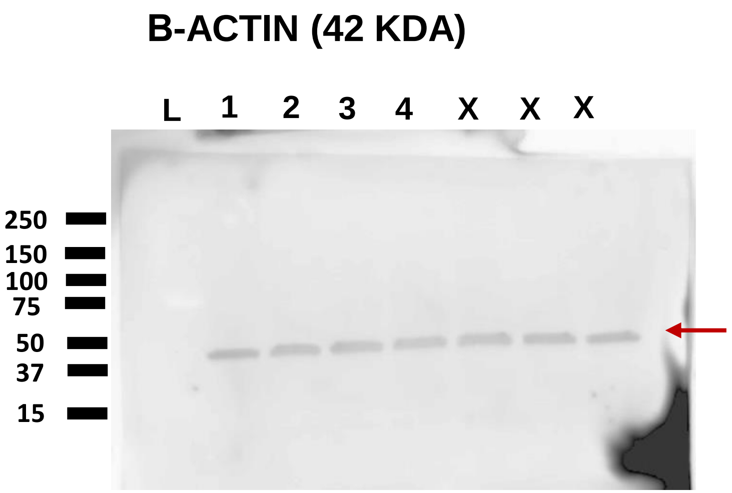

L – ladder
1 – No transfection
2 – *siCD133-1* (10nM)
3 – *siCD133-2* (10nM)
4 – *Scramble* (10nM)

Figure 4A (not pictured)

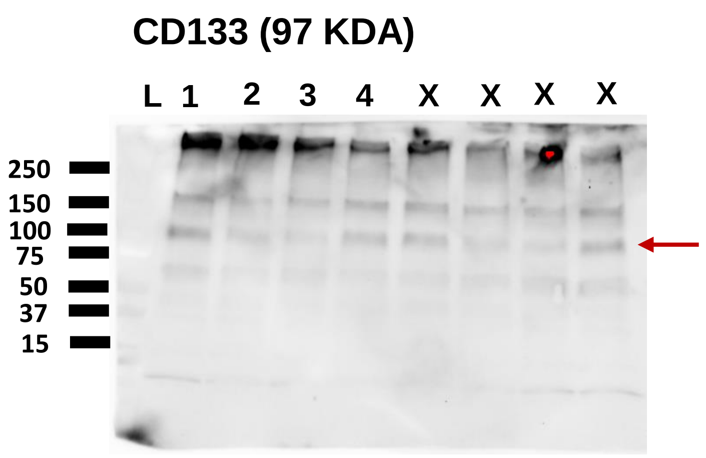

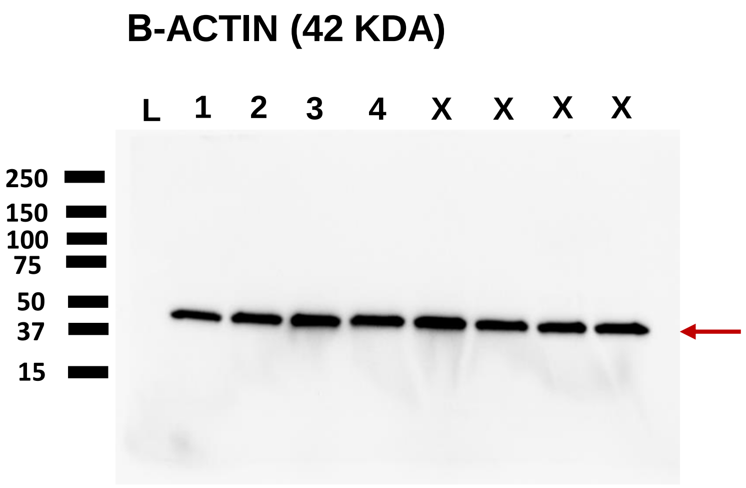

L – ladder
1 – No transfection
2 – *siCD133-1* (10nM)
3 – *siCD133-2* (10nM)
4 – *Scramble* (10nM)

Figure S1D

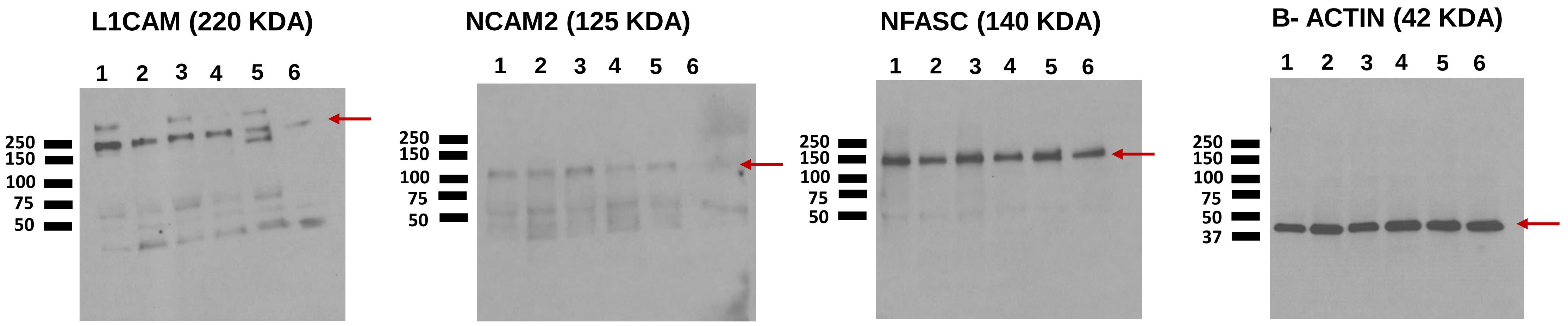

1 – ACM cultured 1
2 – Control 1
3 – ACM cultured 2
4 – Control 2
5 – ACM cultured 3
6 – Control 3
