# Supplementary material for: Astrocytes influence medulloblastoma phenotypes and CD133 surface expression
Source: PLoS One. 2020 Jul 6;15(7):e0235852. doi: 10.1371/journal.pone.0235852 (PMC7337293; doi:10.1371/journal.pone.0235852)
Supplement: Attachment — Submitted filename: Reviewer Response Letter_Gronseth et al_2.1.docx [file pone.0235852.s005.docx]

**REVIEWER RESPONSE LETTER**

Re: PONE-D-20-06629: Astrocytes influence medulloblastoma phenotypes and CD133 surface expression

***Editor Comments:***

Albeit of interest, the paper in its present form requires to be consistently amended in several parts, following all the points highlighted by the two referees. Actually to perform a microarray does not make sense if the altered genes are not investigated and without a confirmation by RT-PCR.

**Response:** We appreciate the concern here. The microarray was performed as a hypothesis-generating experiment. Based on this experiment, we observed that adhesion pathways (Fig. S1B) were influenced in medulloblastoma tumor cells that were conditioned in astrocyte media. Yes, we did validate the targets. In the revision, we have now included the qPCR (Fig. S1C) and western blot (Fig. S1D) results for select microarray targets related to adhesion (NFASC, L1CAM, NCAM2) that were upregulated in ACM-MB cells.

Moreover, the stemness must be observed by means of the specific genes including at least Nanog and OCT3/4.

**Response:** We performed Sox2 western blot (Fig. 3F), which is also a stem marker and found no difference between ACM-conditioned and DMEM-conditioned MB cells. Due to current COVID-19 situation, we are unable to perform other stem marker genes as requested. We have therefore made revisions in the text to de-emphasize the stemness point.

Discussion is largely speculative and long. Therefore, the Authors must amend each point raised in the referee's comments in their revised paper.

**Response:** We have made extensive changes to the discussion to make the points that are directly relevant to the data in the manuscript, and those that support or oppose existing literature on this subject. We provide below a point-by-point response to the reviewer queries.

***Journal Requirements:***

When submitting your revision, we need you to address these additional requirements:

1. Please ensure that your manuscript meets PLOS ONE's style requirements, including those for file naming. The PLOS ONE style templates can be found at http://www.plosone.org/attachments/PLOSOne_formatting_sample_main_body.pdf and http://www.plosone.org/attachments/PLOSOne_formatting_sample_title_authors_affiliations.pdf

**Response:** We have ensured that the manuscript and its file names meet PLOS ONE style requirements.

3. Please amend your current ethics statement to include the full name of the ethics committee that approved your specific study.
For additional information about PLOS ONE submissions requirements for ethics oversight of animal work, please refer to http://journals.plos.org/plosone/s/submission-guidelines#loc-animal-research. Once you have amended this/these statement(s) in the Methods section of the manuscript, please add the same text to the “Ethics Statement” field of the submission form (via “Edit Submission”).

**Response:** The requested changes have been made.

4. We note that you are reporting an analysis of a microarray, next-generation sequencing, or deep sequencing data set. PLOS requires that authors comply with field-specific standards for preparation, recording, and deposition of data in repositories appropriate to their field. Please upload these data to a stable, public repository (such as ArrayExpress, Gene Expression Omnibus (GEO), DNA Data Bank of Japan (DDBJ), NCBI GenBank, NCBI Sequence Read Archive, or EMBL Nucleotide Sequence Database (ENA)). In your revised cover letter, please provide the relevant accession numbers that may be used to access these data. For a full list of recommended repositories, see <http://journals.plos.org/plosone/s/data-availability#loc-omics> or <http://journals.plos.org/plosone/s/data-availability#loc-sequencing>.

**Response:** We plan to submit the microarray data to the GEO database and will provide the reference number upon completion.

5. In your Methods section, please provide additional details regarding the cell lines used in your study. For the UW228/1 cell line, please address whether the cell line was verified, and if so, how it was verified. Please also ensure that you have cited the original paper describing this cell line. For the Daoy cell line, please include the ATCC catalog number. For more information on PLOS ONE's guidelines for research using cell lines, see https://journals.plos.org/plosone/s/submission-guidelines#loc-cell-lines.

**Response:** Short Tandem Repeat (STR) genetic profiling for cell authentication was performed on UW228/1 cells. Although these cells do not have a public STR profile for positive comparison, the STR analysis confirmed no match with any other cell lines within the DSMZ database in addition to confirming a single donor source. This is noted in the revised manuscript.

6. PLOS ONE now requires that authors provide the original uncropped and unadjusted images underlying all blot or gel results reported in a submission’s figures or Supporting Information files. This policy and the journal’s other requirements for blot/gel reporting and figure preparation are described in detail at https://journals.plos.org/plosone/s/figures#loc-blot-and-gel-reporting-requirements and https://journals.plos.org/plosone/s/figures#loc-preparing-figures-from-image-files. When you submit your revised manuscript, please ensure that your figures adhere fully to these guidelines and provide the original underlying images for all blot or gel data reported in your submission. See the following link for instructions on providing the original image data: https://journals.plos.org/plosone/s/figures#loc-original-images-for-blots-and-gels.

**Response:** The requested supporting information is included.

In your cover letter, please note whether your blot/gel image data are in Supporting Information or posted at a public data repository, provide the repository URL if relevant, and provide specific details as to which raw blot/gel images, if any, are not available. Email us at plosone@plos.org if you have any questions.
**Response:** This information has been provided in the supporting information (file “S1_raw_images”).

***Reviewers' comments:***

We thank both reviewers for evaluating our manuscript and providing constructive comments.

**Reviewer #1:**
The image of neurospheres must be changed. The authors must add the pictures with an original magnification grater in order to detect the differences in size following treatments.

**Response:** Yes, we realize this concern but due to COVID-19 related lab closures, hibernations, and other issues, it is unclear when institution will open up. Deliveries are also delayed these days, and thus the May 10 deadline is not feasible. Thus, we are unable to perform additional new experiments to generate spheres for better high power imaging. For better visualization of the spheres, we enlarged the representative images (Fig 3) as much as possible while maintaining good image quality. In terms of size determinations, we have used two independent methods of quantifications for images taken on two different scopes (Zeiss and Keyence), which has been explained in detailed later (rev. 2 query below), and showed the same result. We also excluded spheres less than 75 μm to avoid including aggregates in our quantification. Thus, we are confident with our neurosphere quantification.

Moreover, to confirm the stemness of cells treated with ACM, the authors must evaluate stem transcriptional factors such as OCT4, Nanog and Sox2.

**Response:** We performed Sox2 western blot (Fig. 3F), which is also a stem marker and found no difference between ACM-conditioned and DMEM-conditioned MB cells. Due to current COVID-19 situation, we are unable to perform other stem marker genes as requested. We have therefore made revisions in the text to de-emphasize the stemness point.

The authors demonstrate that CD133 is associated with increased adhesion. As the microarray indicates that adhesion genes are increased, the authors should analyze the genes with the highest value for each experimental condition and evaluate how they change. When CD133 is silenced, what do it happens to these genes?

**Response:** The microarray was performed as a hypothesis-generating experiment. Based on this experiment, we observed that adhesion pathways (Fig. S1B) were influenced in medulloblastoma tumor cells (Daoy) that were conditioned in astrocyte media. Yes, we did validate the targets. In the revision, we have now included the qPCR (Fig. S1C) and western blot (Fig. S1D) results for select adhesion gene targets (*NFASC, L1CAM, NCAM2*) upregulated in ACM-MB cells. We never claimed that the adhesion gene targets *NFASC, L1CAM* and *NCAM2* were downstream of CD133, and thus did not see the rationale for performing this experiment.

The Discussion section must be revised. It is too speculative. It should be better focused on the importance of results obtained in medulloblastoma treatment.

**Response:** We have made extensive changes to the discussion to make the points that are directly relevant to the data in the manuscript, and those that support or oppose existing literature on this subject. Please see revised manuscript with track changes.

**Reviewer #2:**

It is not clear why the Authors performed a microarray, showing a strong alteration of adhesion gene, but did not investigate the role of any of those genes in MB to establish a mechanist effect.

**Response:** We apologize if the microarray rationale was not clear. The microarray was performed as a hypothesis-generating experiment. Based on this experiment, we observed that adhesion pathways (Fig. S1B) were influenced in medulloblastoma (MB) tumor cells that were conditioned in astrocyte media. Yes, we did validate the adhesion gene targets. In the revision, we have now incuded the qPCR (Fig. S1C) and western blot (Fig. S1D) results for select targets (*NFASC, L1CAM, NCAM2*) upregulated in ACM-MB cells. We also performed knockdown of L1CAM target but did not observe changes in adhesion (data not shown). Thus, we rationalized redundancy in the up regulated adhesion molecules, and focused on a known CD133 adhesion molecule that is associated with MB progression [1]. Without the microarray data, we would not have hypothesized that the adhesion pathway was involved in astrocyte conditioned MB cell phenotype. Some of these explanations have been included in the revised MS.

The Author state that ACM increased the stemness of MB cells, however this was based only on spheroid formation. For instance the spheroid analysis and images are not acceptable, more clear and higher magnification images should be provided and an algorithm to calculate sphere diameter should be used.

**Response:** We have now de-emphasized the stemness point, because our Sox2 western blot data showed great variability between DMEM-conditioned MB cells and ACM-conditioned MB cells (Fig. 3F), which precludes any conclusion. We actually performed two independent methods of quantification for images taken under two different scopes. We initially took pictures on Zeiss scope. To measure diameter, we performed a rough calibration of pixels to micrometer (μm), and then used ImageJ for further data processing. In the second Keyence scope, we pre-programmed set points for picture, which removes bias, and used the Keyence analysis software to directly measure micrometer size of the spheres. In this second method, the diameter measurement was more straightforward, and unbiased. Further, we only calculated spheres with greater than 75 μm to avoid including aggregates. Results from both methods showed the same result. In terms of new and better images, we will need to perform the experiment again. Under normal circumstances, we would be more than happy to do it. But, unfortunately, due to COVID-19 associated lab and institution closure, we cannot perform new experiments. For the representative images in Figure 3, we have enlarged our existing pictures (as much as possible while maintaining good image quality) for better visualization of the spheres.

Then, to show an increase in stemness features only spheres formation it’s not enough, the Author should at least show an increase of stem-associated-genes such as Nanog, OCT3/4, and Sox-2 or perform side population assay, or ALDH or any other assay appropriated for MB. This is valid as well for the CD133 knock down.
**Response:** We appreciate the reviewer suggestion, and did perform Sox2 western blot (Fig. 3F), which is also a stem marker [2] and found no difference between ACM-conditioned and DMEM-conditioned MB cells. Due to current COVID-19 situation, we are unable to perform other stem marker genes as requested. We have therefore made revisions in the text to de-emphasize the stemness point.

Discussion should be more focused and less speculative.

**Response:** We have made extensive changes to the discussion to make the points that are directly relevant to the data in the manuscript, and those that support or oppose existing literature on this subject.

We thank the reviewers for spending their valuable time providing us feedback on our manuscript.

**REFERENCES**

1.	Singh SK, Clarke ID, Terasaki M, Bonn VE, Hawkins C, Squire J, et al. Identification of a cancer stem cell in human brain tumors. Cancer Res. 2003;63(18):5821-8. PubMed PMID: 14522905.

2.	Ellis P, Fagan BM, Magness ST, Hutton S, Taranova O, Hayashi S, et al. SOX2, a persistent marker for multipotential neural stem cells derived from embryonic stem cells, the embryo or the adult. Dev Neurosci. 2004;26(2-4):148-65. PubMed PMID: 15711057.
