# Supplementary material for: Astrocytes influence medulloblastoma phenotypes and CD133 surface expression
Source: PLoS One. 2020 Jul 6;15(7):e0235852. doi: 10.1371/journal.pone.0235852 (PMC7337293; doi:10.1371/journal.pone.0235852)
Supplement: Attachment — Submitted filename: Reviewer Response Letter2_Gronseth et al.docx [file pone.0235852.s006.docx]

**REVIEWER RESPONSE LETTER**

Re: PONE-D-20-06629: Astrocytes influence medulloblastoma phenotypes and CD133 surface expression

***Editor Comments:*** The Authors only partly positively responded to the comments previously raised by the two referees. In their answer the Authors wrote that they were unable to perform extra experiments due to the SARS-COV-2 pandemia. This Editor understand that the problem is real but asks the Authors to obtain much more time for research completion. Therefore, the authors are allowed to ask for more time to send their revised manuscript.

Reviewer #1: The Authors do not fully address my previous concerns. They state that, due to current COVID-19 situation, they are unable to perform experiments requested by this reviewer. I recommend asking to editor more time to carry out the experiments in order to improve the manuscript.

Reviewer #2: the author have addressed part of the comments raised by this reviewer, according to author's responce they cannot perform required experiment because of the covid-19 spread and lab closure. Due to this situation I believe it will be more appropriate a time extension so the revising can be performed at the best.

**Response:** Thank you for the additional time that the editor provided to perform additional experiments and make more revisions to the manuscript. As per the reviewer and editor requests, we have now performed additional experiments and included new data in the following panels Figs. 3C, 3D, and 3F.

*New images for neurospheres were requested.* We performed a new experiment to generate newer images under higher power to honor this request. All of our previous images were low power. This new higher magnified image data is now included in figure 3C with scale bars. The neurosphere sizes are clearly larger in the astrocyte-conditioned media (ACM) compared to DMEM conditions. Further, the ACM-induced neurospheres are similar in size to DMEM:F12 positive control conditions. The new images were quantified, and the quantification is also provided in the figure 3D.

*Western blots for additional stem cell proteins (Nanog & Oct4) were requested* in addition to previously provided Nestin (Fig. 3E) and Sox2 (Fig. 3G) data. We performed westerns for Oct-4 and Nanog, and have provided new data for Oct4 (Fig. 3F) along with quantification. The Oct-4A blot shows that under both ACM and DMEM:F12 positive control conditions, Oct-4A protein (***p<0.001) expression was higher compared to DMEM conditions. We checked Nanog protein as well, but the antibody did not work well, and thus no conclusion can be further derived from this blot. Collectively, of the three stemness proteins that were detected in MB cells, we noticed 2 (Nestin and Oct-4A) out of 3 expression to be higher in ACM conditions compared to DMEM conditions.

*Stemness write-up de-emphasis.* We carefully searched for the entire manuscript for the stemness or stem cell word, and only found that we utilized these words in reference to describing CD133 as a stem cell marker or describing Nestin, Sox2 or Oct-4A proteins in the results section. The Discussion section was extensively revised in the previous submission, and all references to stemness was removed then.
